# Crystal structure of *N*,*N*-di­ethyl­benzene-1,4-diaminium dinitrate

**DOI:** 10.1107/S1600536814022946

**Published:** 2014-10-24

**Authors:** Yasmina Bouaoud, Graham Smith, Hocine Merazig, Zouaoui Setifi

**Affiliations:** a Unité de Recherche de Chimie de l’Environnement et Moléculaire Structurale (CHEMS), Université Constantine 1, Constantine 25000, Algeria; b Science and Engineering Faculty, Queensland University of Technology, GPO Box 2434, Brisbane, Queensland 4001, Australia; c Département de Technologie, Faculté de Technologie, Université 20 Août 1955-Skikda, BP 26, Route d’El-Hadaiek, Skikda 21000, Algeria

**Keywords:** crystal structure, diaminium, nitrate salt, hydrogen bonding

## Abstract

In the structure of the title mol­ecular salt, C_10_H_18_N_2_^2+^·2NO_3_^−^, the dinitrate salt of 4-(*N*,*N*-di­ethyl­amino)­aniline, the two ethyl groups lie almost perpendicular to the plane of the benzene ring [the ring-to-ethyl C—C—N—C torsion angles are −59.5 (2) and 67.5 (3)°]. The aminium groups of the cation form inter-species N—H⋯O hydrogen bonds with the nitro O-atom acceptors of both anions, giving rise to chain substructures lying along *c*. The chains are linked *via* further N—H⋯O hydrogen bonds, forming two-dimensional networks lying parallel to (010). These sheets are linked by C—H⋯O hydrogen bonds, forming a three-dimensional structure.

## Related literature   

For the structures of metal complex structures with dicationic 4-[*N,N*-di­ethyl­amino)­aniline or 4-[*N,N*-di­ethyl­amino)-2-methyl­aniline species as counter-ions, see: Dobrzycki & Woźniak (2008 ▶); Bringley *et al.* (2005 ▶). For the structure of similar dicationic benzene-1,4-diaminium species, see: Chandrasekaran (1969 ▶); Anderson *et al.* (2006 ▶).
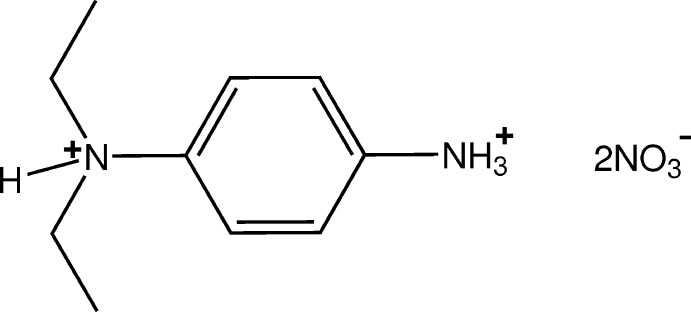


## Experimental   

### Crystal data  

C_10_H_18_N_2_^2+^·2NO_3_^−^
*M* *_r_* = 290.28
Orthorhombic, 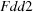

*a* = 38.821 (5) Å
*b* = 20.900 (5) Å
*c* = 7.172 (5) Å
*V* = 5819 (4) Å^3^
*Z* = 16
Mo *K*α radiation
μ = 0.11 mm^−1^
*T* = 293 K
0.30 × 0.18 × 0.09 mm

### Data collection  

Bruker APEXII CCD diffractometer
Absorption correction: multi-scan (*SADABS*; Sheldrick, 2003 ▶) *T*_min_ = 0.950, *T*_max_ = 0.988
7645 measured reflections
3156 independent reflections
2522 reflections with *I* > 2σ(*I*)
*R*_int_ = 0.046

### Refinement  

*R*[*F* ^2^ > 2σ(*F* ^2^)] = 0.043
*wR*(*F* ^2^) = 0.111
*S* = 1.03
3156 reflections
193 parameters
5 restraints
H atoms treated by a mixture of independent and constrained refinement
Δρ_max_ = 0.17 e Å^−3^
Δρ_min_ = −0.19 e Å^−3^

### 

Data collection: *APEX2* (Bruker, 2009 ▶); cell refinement: *APEX2* and *SAINT* (Bruker, 2009 ▶); data reduction: *SAINT*; program(s) used to solve structure: *SHELXS97* (Sheldrick, 2008 ▶); program(s) used to refine structure: *SHELXL97* (Sheldrick, 2008 ▶) within *WinGX* (Farrugia, 2012 ▶); molecular graphics: *PLATON* (Spek, 2009 ▶); software used to prepare material for publication: *PLATON*.

## Supplementary Material

Crystal structure: contains datablock(s) global, I. DOI: 10.1107/S1600536814022946/su5003sup1.cif

Structure factors: contains datablock(s) I. DOI: 10.1107/S1600536814022946/su5003Isup2.hkl

Supporting information file. DOI: 10.1107/S1600536814022946/su5003Isup3.cml

**Fig. 1.**
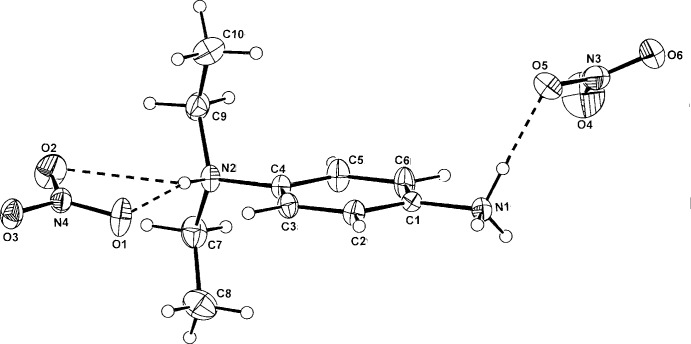
The molecular structure of the title salt, with atom labelling. Displacement ellipsoids are drawn at the 30% probability level. Hydrogen bonds are shown as dashed lines (see Table 1 for details).

**Fig. 2.**
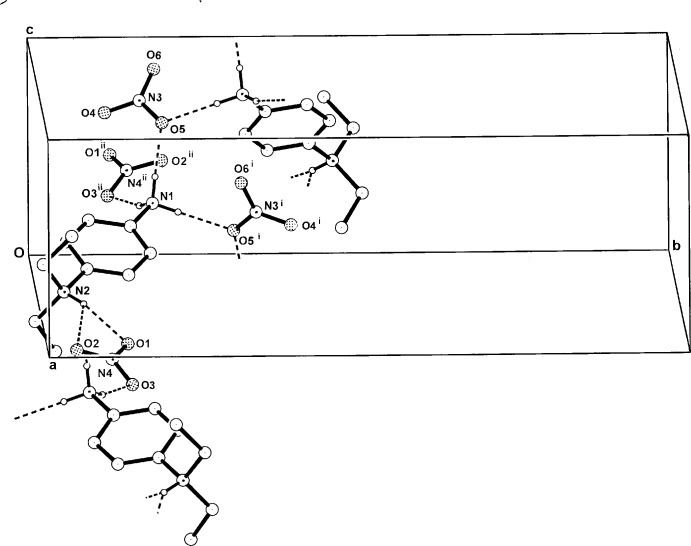
A partial extension of the cation–anion chain substructure in the title salt in the unit cell viewed along a. Non-associative H-atoms are omitted and formal hydrogen-bonding associations are shown as dashed lines (see Table 1 for details; for symmetry codes see Table 1).

**Fig. 3.**
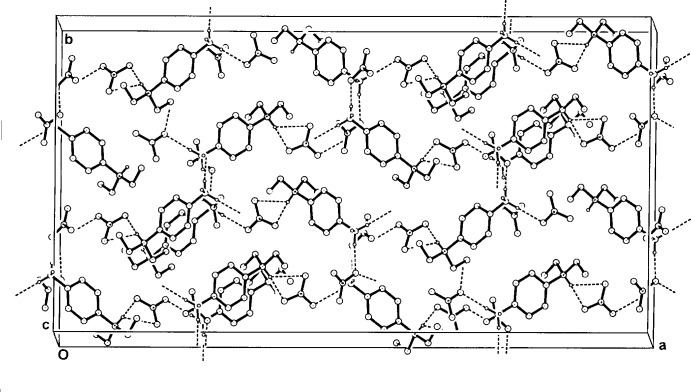
The crystal packing of the title salt viewed along c, illustrating the three-dimensional structure. Hydrogen bonds are shown as dashed lines (see Table 1 for details).

. DOI: 10.1107/S1600536814022946/su5003fig1.tif
The mol­ecular structure of the title salt, with atom labelling. Displacement ellipsoids are drawn at the 30% probability level. Hydrogen bonds are shown as dashed lines (see Table 1 for details).

a . DOI: 10.1107/S1600536814022946/su5003fig2.tif
A partial extension of the cation–anion chain substructure in the title salt in the unit cell viewed along *a*. Non-associative H-atoms are omitted and formal hydrogen-bonding associations are shown as dashed lines (see Table 1 for details; for symmetry codes see Table 1).

c . DOI: 10.1107/S1600536814022946/su5003fig3.tif
The crystal packing of the title salt viewed along *c*, illustrating the three-dimensional structure. Hydrogen bonds are shown as dashed lines (see Table 1 for details).

CCDC reference: 1029930

Additional supporting information:  crystallographic information; 3D view; checkCIF report

## Figures and Tables

**Table 1:** Hydrogen-bond geometry (, )

*D*H*A*	*D*H	H*A*	*D* *A*	*D*H*A*
N1H1*A*O5	0.90(2)	1.84(2)	2.731(3)	178(2)
N1H1*B*O5^i^	0.89(2)	1.92(2)	2.803(3)	175(2)
N1H1*C*O3^ii^	0.89(2)	1.99(2)	2.855(3)	165(2)
N2H21O1	0.87(2)	2.08(2)	2.930(3)	167(2)
N2H21O2	0.87(2)	2.60(2)	3.198(3)	127(2)
C2H2O2^iii^	0.93	2.47	3.216(4)	137
C3H3O1	0.93	2.46	3.236(4)	141
C5H5O3^iv^	0.93	2.57	3.467(4)	161
C8H8*A*O3^iv^	0.97	2.59	3.552(4)	173

Symmetry codes: (i) 
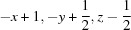
; (ii) 
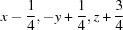
; (iii) 
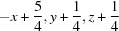
; (iv) 
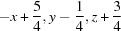
.

## supplementary crystallographic information

### S1. Comment

In the Cambridge Structural Database (CSD, V5.35, last update May 2014; Allen, 2002), only one structure with the title dicationic species 4-(N,N-diethylamino)aniline (DEAA^2+^) as a counter-ion is found, in the complex (DEAA^2+^)_2_ [PbCl_6_]^4-^ hydrate (Dobrzycki & Woźniak, 2008). Complexes with the analogous 4-(*N,N*-diethylamino)-2-methylaniline dication as a counter-ion are also known, *e.g.* with [CuCl_4_]^2-^ (Dobrzycki & Woźniak, 2008) and with [Ag_2_Br_6_]^4-^ and [Ag_2_I_6_]^4-^ (Bringley *et al.*, 2005).

In the title compound, Fig. 1, the two ethyl groups lie almost perpendicular to the plane of the benzene ring [C5—C4—N2—C7/C8 torsion angles are 67.5 (3) and -59.5 (2)°], respectively, which is similar to the conformation assumed by these groups in the structures of the analogous dications in all of the prevously mentioned complex structures.

In the crystal of the title salt, all the aminium groups of the cation form inter-species N—H···O hydrogen bonds with the nitro O-atoms of both anions (Table 1), which includes an asymmetric three-centre *R*^2^_1_(4) association with the tertiary aminium group (N2) and O1 and O2 (Table 1). One-dimensional chains are formed along *c* (Fig. 2) and are further extended into a two-dimensional network lying parallel to (010). Weak C—H···O hydrogen-bonding associations are also present in an overall three-dimensional structure (Fig. 3), in which nitrate O-atoms O4 and O6 are not involved in any interactions.

### S2. Experimental

The title compound was synthesized from a mixture of Ni(NO_3_)_2_. 6H_2_O (291 mg, 1 mmol) and 4-(*N,N*-diethylamino)aniline sulfate (262 mg, 1 mmol) in methanol (40 ml). The resulting solution was stirred for 30 min at room temperature. After 10 d, single crystals suitable for X-ray diffraction were collected by filtration, washed with water and dried in air (yield 35%).

### S3. Refinement

N-bound H atoms were located from a difference Fourier map and included in the refinement with restraints [N—H = 0.88 (2) Å] and allowed to ride with *U*_iso_(H) = 1.2*U*_eq_(N). Other H-atoms were placed in calculated positions [C—H = 0.93 Å (aromatic), 0.97 Å (methylene) and 0.96 Å (methyl)] and allowed to ride in the refinement, with *U*_iso_(H) = 1.2*U*_eq_(C) (aromatic and methylene) or 1.5*U*_eq_(C) (methyl).

### Crystal data

**Table:**

C_10_H_18_N_2_^2+^·2NO_3_^−^	*F*(000) = 2464
*M**_r_* = 290.28	*D*_x_ = 1.325 Mg m^−^^3^
Orthorhombic, *F**d**d*2	Mo *K*α radiation, λ = 0.71073 Å
Hall symbol: F 2 -2d	Cell parameters from 6123 reflections
*a* = 38.821 (5) Å	θ = 2.4–30.4°
*b* = 20.900 (5) Å	µ = 0.11 mm^−^^1^
*c* = 7.172 (5) Å	*T* = 293 K
*V* = 5819 (4) Å^3^	Plate, brown
*Z* = 16	0.30 × 0.18 × 0.09 mm

### Data collection

**Table:**

Bruker APEXII CCD diffractometer	3156 independent reflections
Radiation source: fine-focus sealed tube	2522 reflections with *I* > 2σ(*I*)
Graphite monochromator	*R*_int_ = 0.046
ω–2θ scans	θ_max_ = 27.4°, θ_min_ = 2.9°
Absorption correction: multi-scan (*SADABS*; Sheldrick, 2003)	*h* = −50→42
*T*_min_ = 0.950, *T*_max_ = 0.988	*k* = −21→26
7645 measured reflections	*l* = −8→9

### Refinement

**Table:**

Refinement on *F*^2^	Primary atom site location: structure-invariant direct methods
Least-squares matrix: full	Secondary atom site location: difference Fourier map
*R*[*F*^2^ > 2σ(*F*^2^)] = 0.043	Hydrogen site location: inferred from neighbouring sites
*wR*(*F*^2^) = 0.111	H atoms treated by a mixture of independent and constrained refinement
*S* = 1.03	*w* = 1/[σ^2^(*F*_o_^2^) + (0.0471*P*)^2^ + 2.0648*P*] where *P* = (*F*_o_^2^ + 2*F*_c_^2^)/3
3156 reflections	(Δ/σ)_max_ = 0.001
193 parameters	Δρ_max_ = 0.17 e Å^−^^3^
5 restraints	Δρ_min_ = −0.19 e Å^−^^3^

### Special details

**Table:**

Geometry. Bond distances, angles *etc*. have been calculated using the rounded fractional coordinates. All su's are estimated from the variances of the (full) variance-covariance matrix. The cell e.s.d.'s are taken into account in the estimation of distances, angles and torsion angles
Refinement. Refinement of *F*^2^ against ALL reflections. The weighted *R*-factor *wR* and goodness of fit *S* are based on *F*^2^, conventional *R*-factors *R* are based on *F*, with *F* set to zero for negative *F*^2^. The threshold expression of *F*^2^ > σ(*F*^2^) is used only for calculating *R*-factors(gt) *etc*. and is not relevant to the choice of reflections for refinement. *R*-factors based on *F*^2^ are statistically about twice as large as those based on *F*, and *R*- factors based on ALL data will be even larger.

### Fractional atomic coordinates and isotropic or equivalent isotropic displacement parameters (Å^2^)

**Table:**

	*x*	*y*	*z*	*U*_iso_*/*U*_eq_	
N1	0.49457 (4)	0.17907 (9)	0.4684 (3)	0.0325 (5)	
N2	0.59991 (4)	0.03665 (8)	0.1133 (3)	0.0347 (5)	
C1	0.52168 (4)	0.14297 (9)	0.3774 (3)	0.0279 (5)	
C2	0.54483 (5)	0.17368 (10)	0.2631 (3)	0.0326 (6)	
C3	0.57043 (5)	0.13844 (10)	0.1755 (3)	0.0334 (6)	
C4	0.57233 (4)	0.07369 (9)	0.2053 (3)	0.0298 (6)	
C5	0.54930 (5)	0.04263 (10)	0.3202 (3)	0.0438 (7)	
C6	0.52374 (5)	0.07787 (10)	0.4069 (3)	0.0427 (7)	
C7	0.58608 (6)	−0.00904 (11)	−0.0298 (4)	0.0496 (8)	
C8	0.62331 (6)	0.00310 (12)	0.2507 (4)	0.0482 (8)	
C9	0.56544 (8)	0.02374 (14)	−0.1775 (4)	0.0669 (10)	
C10	0.63942 (7)	0.04803 (16)	0.3874 (5)	0.0715 (10)	
O1	0.63464 (4)	0.13421 (8)	−0.1120 (3)	0.0636 (6)	
O2	0.66914 (4)	0.05414 (9)	−0.1229 (3)	0.0650 (7)	
O3	0.68100 (4)	0.13959 (8)	−0.2769 (2)	0.0463 (5)	
N4	0.66175 (4)	0.10900 (9)	−0.1699 (2)	0.0370 (5)	
O4	0.47981 (6)	0.10514 (10)	0.8776 (4)	0.1004 (10)	
O5	0.50571 (4)	0.19404 (7)	0.8419 (2)	0.0464 (5)	
O6	0.47157 (5)	0.18295 (13)	1.0743 (3)	0.0839 (8)	
N3	0.48499 (5)	0.15919 (11)	0.9348 (3)	0.0503 (7)	
H1A	0.4986 (5)	0.1832 (10)	0.591 (2)	0.0390*	
H1B	0.4959 (5)	0.2192 (8)	0.428 (3)	0.0390*	
H1C	0.4749 (4)	0.1587 (10)	0.448 (3)	0.0390*	
H2	0.54330	0.21760	0.24480	0.0390*	
H3	0.58620	0.15850	0.09710	0.0400*	
H5	0.55090	−0.00130	0.33920	0.0530*	
H6	0.50800	0.05770	0.48500	0.0510*	
H7A	0.60520	−0.03140	−0.08780	0.0600*	
H7B	0.57170	−0.04060	0.03180	0.0600*	
H8A	0.61010	−0.02880	0.31810	0.0580*	
H8B	0.64140	−0.01890	0.18270	0.0580*	
H9A	0.55700	−0.00740	−0.26470	0.1010*	
H9B	0.57970	0.05410	−0.24160	0.1010*	
H9C	0.54630	0.04560	−0.12110	0.1010*	
H10A	0.65400	0.02450	0.47070	0.1070*	
H10B	0.62170	0.06920	0.45760	0.1070*	
H10C	0.65280	0.07930	0.32170	0.1070*	
H21	0.6122 (5)	0.0669 (9)	0.063 (3)	0.0420*	

### Atomic displacement parameters (Å^2^)

**Table:**

	*U*^11^	*U*^22^	*U*^33^	*U*^12^	*U*^13^	*U*^23^
N1	0.0309 (8)	0.0341 (10)	0.0325 (9)	−0.0016 (7)	0.0053 (7)	−0.0021 (8)
N2	0.0328 (8)	0.0319 (9)	0.0393 (10)	0.0006 (7)	0.0112 (8)	−0.0013 (8)
C1	0.0254 (8)	0.0315 (10)	0.0269 (10)	0.0005 (7)	0.0000 (7)	−0.0008 (9)
C2	0.0324 (9)	0.0246 (10)	0.0407 (11)	−0.0032 (8)	0.0056 (9)	0.0027 (8)
C3	0.0310 (9)	0.0332 (10)	0.0360 (11)	−0.0076 (8)	0.0083 (8)	0.0025 (9)
C4	0.0285 (9)	0.0274 (10)	0.0336 (10)	0.0003 (8)	0.0060 (8)	−0.0021 (9)
C5	0.0471 (12)	0.0260 (11)	0.0582 (14)	0.0007 (9)	0.0193 (11)	0.0061 (10)
C6	0.0411 (11)	0.0346 (12)	0.0523 (14)	−0.0042 (9)	0.0210 (10)	0.0096 (11)
C7	0.0531 (12)	0.0396 (13)	0.0562 (15)	−0.0013 (10)	0.0095 (12)	−0.0171 (12)
C8	0.0400 (11)	0.0537 (15)	0.0510 (13)	0.0125 (10)	0.0069 (11)	0.0077 (12)
C9	0.0802 (17)	0.068 (2)	0.0525 (15)	−0.0061 (15)	−0.0071 (15)	−0.0158 (16)
C10	0.0478 (13)	0.099 (2)	0.0676 (18)	0.0045 (14)	−0.0118 (14)	0.0000 (18)
O1	0.0462 (9)	0.0616 (11)	0.0829 (13)	0.0066 (8)	0.0289 (10)	−0.0033 (11)
O2	0.0620 (10)	0.0481 (10)	0.0850 (14)	0.0088 (8)	0.0077 (11)	0.0312 (11)
O3	0.0395 (8)	0.0460 (9)	0.0534 (10)	0.0014 (7)	0.0143 (7)	0.0140 (8)
N4	0.0318 (8)	0.0412 (10)	0.0380 (10)	−0.0007 (8)	0.0055 (8)	−0.0010 (9)
O4	0.1129 (17)	0.0573 (13)	0.131 (2)	−0.0358 (12)	0.0083 (18)	0.0035 (16)
O5	0.0603 (9)	0.0431 (9)	0.0359 (8)	−0.0042 (7)	0.0010 (8)	−0.0006 (8)
O6	0.0538 (10)	0.156 (2)	0.0418 (10)	0.0075 (12)	0.0096 (9)	−0.0029 (14)
N3	0.0427 (10)	0.0652 (14)	0.0429 (11)	−0.0016 (10)	−0.0053 (9)	0.0083 (11)

### Geometric parameters (Å, º)

**Table:**

O1—N4	1.248 (2)	C4—C5	1.378 (3)
O2—N4	1.229 (3)	C5—C6	1.383 (3)
O3—N4	1.247 (2)	C7—C9	1.495 (4)
O4—N3	1.219 (3)	C8—C10	1.495 (4)
O5—N3	1.273 (3)	C2—H2	0.9300
O6—N3	1.233 (3)	C3—H3	0.9300
N1—C1	1.450 (3)	C5—H5	0.9300
N2—C4	1.477 (3)	C6—H6	0.9300
N2—C8	1.513 (3)	C7—H7A	0.9700
N2—C7	1.501 (3)	C7—H7B	0.9700
N1—H1B	0.889 (17)	C8—H8A	0.9700
N1—H1C	0.886 (17)	C8—H8B	0.9700
N1—H1A	0.897 (15)	C9—H9B	0.9600
N2—H21	0.87 (2)	C9—H9A	0.9600
C1—C2	1.375 (3)	C9—H9C	0.9600
C1—C6	1.379 (3)	C10—H10C	0.9600
C2—C3	1.387 (3)	C10—H10A	0.9600
C3—C4	1.372 (3)	C10—H10B	0.9600
			
C4—N2—C7	112.31 (15)	C1—C2—H2	120.00
C4—N2—C8	112.79 (19)	C3—C2—H2	120.00
C7—N2—C8	111.43 (17)	C4—C3—H3	120.00
H1A—N1—H1B	102.6 (19)	C2—C3—H3	120.00
H1A—N1—H1C	111.0 (19)	C4—C5—H5	121.00
H1B—N1—H1C	116.7 (18)	C6—C5—H5	120.00
C1—N1—H1B	107.6 (13)	C5—C6—H6	120.00
C1—N1—H1C	107.5 (13)	C1—C6—H6	120.00
C1—N1—H1A	111.4 (13)	C9—C7—H7A	109.00
C4—N2—H21	101.6 (13)	N2—C7—H7A	109.00
C7—N2—H21	112.0 (14)	N2—C7—H7B	109.00
C8—N2—H21	106.1 (13)	C9—C7—H7B	109.00
O2—N4—O3	120.47 (17)	H7A—C7—H7B	108.00
O1—N4—O3	119.57 (18)	N2—C8—H8B	109.00
O1—N4—O2	119.96 (17)	N2—C8—H8A	109.00
O4—N3—O5	117.3 (2)	C10—C8—H8B	109.00
O5—N3—O6	117.5 (2)	H8A—C8—H8B	108.00
O4—N3—O6	125.2 (2)	C10—C8—H8A	109.00
C2—C1—C6	120.92 (18)	H9A—C9—H9B	109.00
N1—C1—C6	119.10 (17)	H9A—C9—H9C	109.00
N1—C1—C2	119.98 (17)	C7—C9—H9A	110.00
C1—C2—C3	119.36 (19)	C7—C9—H9B	109.00
C2—C3—C4	119.44 (19)	C7—C9—H9C	110.00
N2—C4—C3	119.10 (17)	H9B—C9—H9C	109.00
C3—C4—C5	121.52 (18)	H10B—C10—H10C	109.00
N2—C4—C5	119.37 (17)	C8—C10—H10A	109.00
C4—C5—C6	118.90 (19)	C8—C10—H10B	109.00
C1—C6—C5	119.86 (19)	C8—C10—H10C	109.00
N2—C7—C9	112.6 (2)	H10A—C10—H10B	109.00
N2—C8—C10	112.8 (2)	H10A—C10—H10C	110.00
			
C7—N2—C4—C3	−113.1 (2)	C6—C1—C2—C3	0.5 (3)
C7—N2—C4—C5	67.5 (3)	N1—C1—C6—C5	179.57 (19)
C8—N2—C4—C3	120.0 (2)	C2—C1—C6—C5	−0.3 (3)
C8—N2—C4—C5	−59.5 (2)	C1—C2—C3—C4	−0.4 (3)
C4—N2—C7—C9	57.5 (3)	C2—C3—C4—N2	−179.28 (19)
C8—N2—C7—C9	−174.8 (2)	C2—C3—C4—C5	0.2 (3)
C4—N2—C8—C10	−56.5 (3)	N2—C4—C5—C6	179.47 (19)
C7—N2—C8—C10	176.1 (2)	C3—C4—C5—C6	0.0 (3)
N1—C1—C2—C3	−179.37 (19)	C4—C5—C6—C1	0.0 (3)

### Hydrogen-bond geometry (Å, º)

**Table:**

*D*—H···*A*	*D*—H	H···*A*	*D*···*A*	*D*—H···*A*
N1—H1*A*···O5	0.90 (2)	1.84 (2)	2.731 (3)	178 (2)
N1—H1*B*···O5^i^	0.89 (2)	1.92 (2)	2.803 (3)	175 (2)
N1—H1*C*···O3^ii^	0.89 (2)	1.99 (2)	2.855 (3)	165 (2)
N2—H21···O1	0.87 (2)	2.08 (2)	2.930 (3)	167 (2)
N2—H21···O2	0.87 (2)	2.60 (2)	3.198 (3)	127 (2)
C2—H2···O2^iii^	0.93	2.47	3.216 (4)	137
C3—H3···O1	0.93	2.46	3.236 (4)	141
C5—H5···O3^iv^	0.93	2.57	3.467 (4)	161
C8—H8*A*···O3^iv^	0.97	2.59	3.552 (4)	173

Symmetry codes: (i) −*x*+1, −*y*+1/2, *z*−1/2; (ii) *x*−1/4, −*y*+1/4, *z*+3/4; (iii) −*x*+5/4, *y*+1/4, *z*+1/4; (iv) −*x*+5/4, *y*−1/4, *z*+3/4.
